# RT-QuIC detection of tauopathies using full-length tau substrates

**DOI:** 10.1080/19336896.2020.1832946

**Published:** 2020-11-10

**Authors:** Joanne M. Tennant, Davin M. Henderson, Thomas M. Wisniewski, Edward A. Hoover

**Affiliations:** a Prion Research Center, Department of Microbiology, Immunology, and Pathology, College of Veterinary Medicine and Biomedical Sciences, Colorado State University, Fort Collins, CO, USA; b Center for Cognitive Neurology and Departments of Neurology, Pathology and Psychiatry, NYU School of Medicine, New York, NY, USA

**Keywords:** Tau, tauopathy, RT-QuIC, Alzheimer’s Disease

## Abstract

Early detection and diagnosis of neurodegenerative diseases has been hampered by the lack of sensitive testing. Real-time quaking induced conversion (RT-QuIC) has been used for the early and sensitive detection of prion-induced neurologic disease, and has more recently been adapted to detect misfolded alpha-synuclein and tau as biomarkers for neurodegenerative disease. Here we use full-length recombinant tau substrates to detect tau seeding activity in Alzheimer’s disease and other human tauopathies.

## Introduction

Early diagnosis of neurodegenerative disease is hampered by a lack of sensitive and specific assays [1]. Biomarkers of neurogenerative diseases, such as the tau protein, are potential substrates for early detection of disease [2]. Misfolded tau has been identified in paired helical filaments (PHFs) and neurofibrillary tangles (NFTs) in Alzheimer’s disease and other neurodegenerative diseases [3,4]. Owing to differential mRNA splicing of the *MAPT* gene, tau can exist as either 0, 1, or 2 N-terminal domains (N) and either 3 or 4 repeats in the carboxy terminal of the protein [5]. The tau repeat domains are associated with microtubule binding in its normal and misfolded amyloid states [6]. The misfolding of tau is also associated with its phosphorylation, which is another diagnostic correlate of AD, and other tauopathies [7].

Tauopathies are classified based on the predominant tau isoform present in paired helical filaments (PHFs), e.g. Pick’s disease is characterized by 3-repeat (3 R) tau isoforms, whereas progressive supranuclear palsy (PSP) and some frontotemporal lobe dementias (FTLDs) are characterized by 4 repeat (4 R) tau isoforms. Alzheimer’s disease and chronic traumatic encephalopathy (CTE) contain a mixture of 3 R and 4 R tau isoforms in the PHFs (3 R/4 R tauopathies) [8,9].

Sensitive detection of misfolded prion protein has been made possible using amplification assays, such as protein misfolding cyclic amplification (PMCA) and real-time quaking induced conversion (RT-QuIC) [10,11] to demonstrate seeding activity in brain, clinically accessible peripheral tissues, body fluids, and excreta [12–14]. Adaptations of RT-QuIC have recently also some synucleinopathies and tauopathies in humans [15–22]. Nevertheless, the sensitive detection of tau misfolding remains difficult due in part to the innate complexity of tau isoforms [20,22,23].

Here we describe detection of the single (3 R or 4 R) and mixed (3 R/4 R) human tauopathies, and P301S transgenic mice using a mixture of purified full-length tau protein containing targeted cysteine to serine substitutions to allow greater sensitivity.

## Results

### Determination of tau seeding activity range

**Figure 1.**
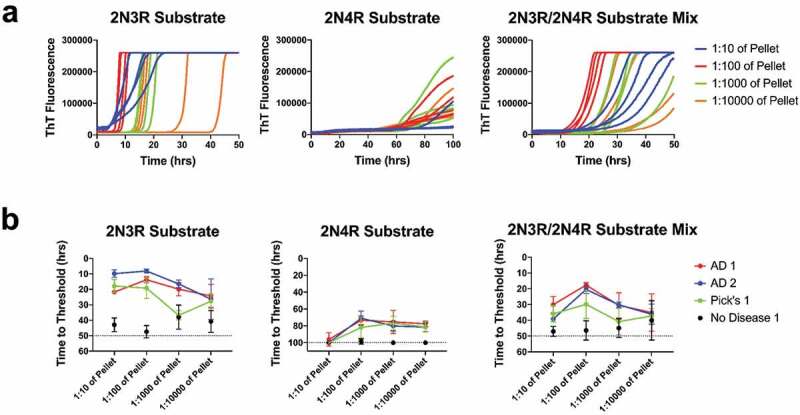
Tau seeding range. (a) Representative ThT fluorescence curves for AD1 brain over 10-fold dilutions of the pellet seeded onto either 2N3R, 2N4R, or both 2N3R/2N4R recombinant tau substrates. Each dilution has four replicates represented. (b) Plotted hours to threshold for 2 separate AD, one Pick’s disease brain, and one no disease control brain over 10-fold dilutions of 2N3R, 2N4R, or 2N3R/2N4R mixed substrate

We purified 2N3R and 2N4R recombinant tau substrates and assessed seeding activity in Alzheimer’s Disease (AD) brain samples using either 2N3R only, 2N4R only, or as a 50:50 2N3R and 2N4R molar ratio mixture (Figure 1(a)). We observed the most consistent seeding activity in ethanol-precipitated AD brain homogenate pellets reconstituted with N2 in PBS vs. non-diseased control brains (Figure 1(a,b)). We also noted greater divergence between diseased vs. non-diseased brain seeding activity as the samples were diluted 10- to 1000-fold, beyond, which seeding activity was no longer statistically significant (Figure 1(b), data not shown). Based on these results, we chose to use a 1:100 dilution of brain homogenate pellets for subsequent testing with 2N3R, 2N4R, or mixed 2N3R/2N4R r-tau substrates.

### Detection of tauopathies with 2N3R substrate

**Figure 2.**
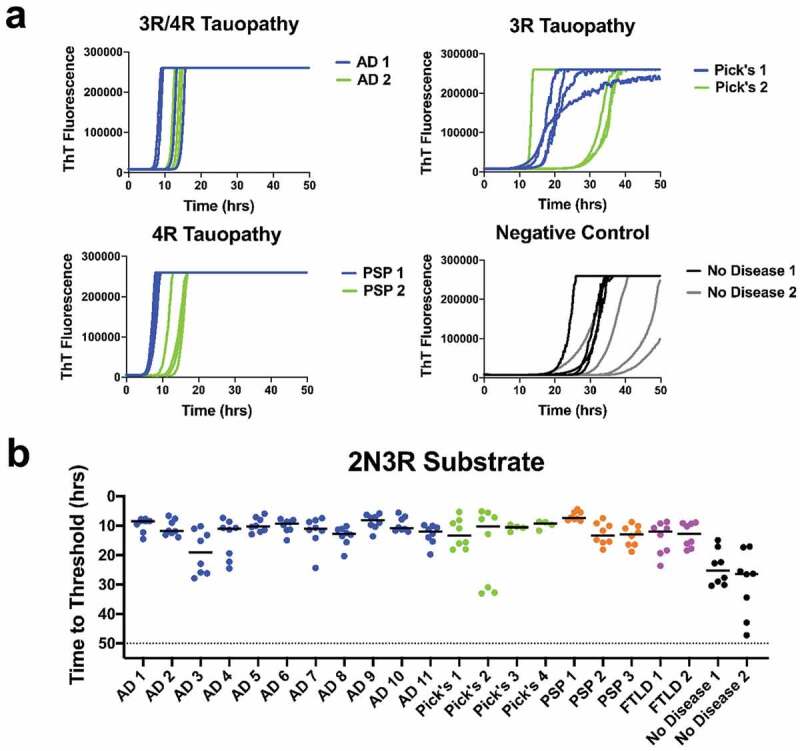
Tauopathy detection using 2N3R substrate: (a) ThT fluorescence curves for AD, Pick’s, and PSP diseased brains as well as 2 no disease brain controls. (b) Time to threshold for 11 AD, 4 Pick’s, 3 PSP, and 2 FTLD disease brains, and 2 no disease controls. All diseased brain samples were statistically different from the negative controls. Sensitivity is good for 3 R and even 4 R disease, but control brain background is high

We evaluated tau seeding activity in 20 tauopathy brains and 2 non-diseased age-matched control brains. Seeding activity for most tauopathy disease samples was greater than negative controls (*p* < 0.05 to *p* < 0.0001), with the Pick’s and Alzheimer’s disease brain samples crossing threshold the earliest (Figure 2(b)). However, we did observe seeding activity in the FTLD and PSP brains, a result unexpected for 3 R only substrate. It is important to note that the non-disease control brain samples also crossed threshold, but at a statistically significantly longer time period (Figure 2(b)).

### Detection of tauopathies with 2N4R substrate

**Figure 3.**
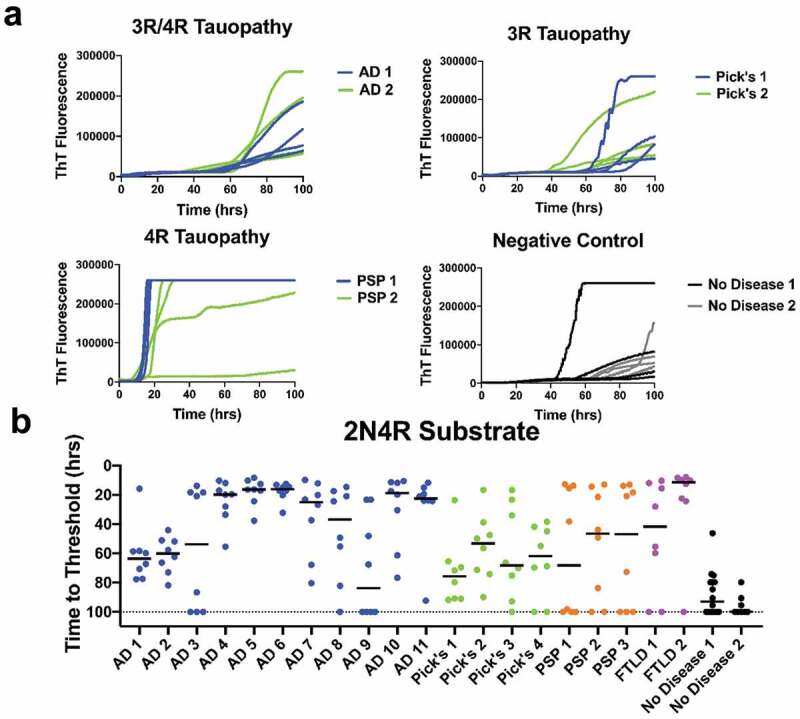
Tauopathy detection with 2N4R substrate. (a) ThT fluorescence curves for AD, Pick’s, PSP, and no disease brain controls in 2N4R substrate. (b) Time to threshold for 11 AD, 4 Pick’s, 3 PSP, 2 FTLD brains and age-appropriate no-disease controls in 2N4R only substrate. Eight replicates for each sample are shown. Detection signals are lengthened and reduced for mixed ((3 R/4 R)(AD) and 3 R (PiD) diseases

Evaluation of tau seeding activity of 22 brain samples was performed using 2N4R substrate. With all other reaction conditions remaining unchanged, the 2N4R substrate was slower to convert to amyloid conformations than was the 2N3R substrate (Figures 2(b) and 3(b)). Extending the assay time to 100 hours was required to discern seeding activities (Figure 3(a,b)). Also noted were that the variance among sample replicates was greater for the 2N4R vs. 2N3R substrate, and that some tauopathy brain samples, e.g. AD3, AD9, and PSP1, were not significantly different (*p* > 0.05) from the no-disease brains (Figures 2(b) and 3(b)).

### Detection of tauopathies using 2N3R/2N4R mixed substrate

**Figure 4.**
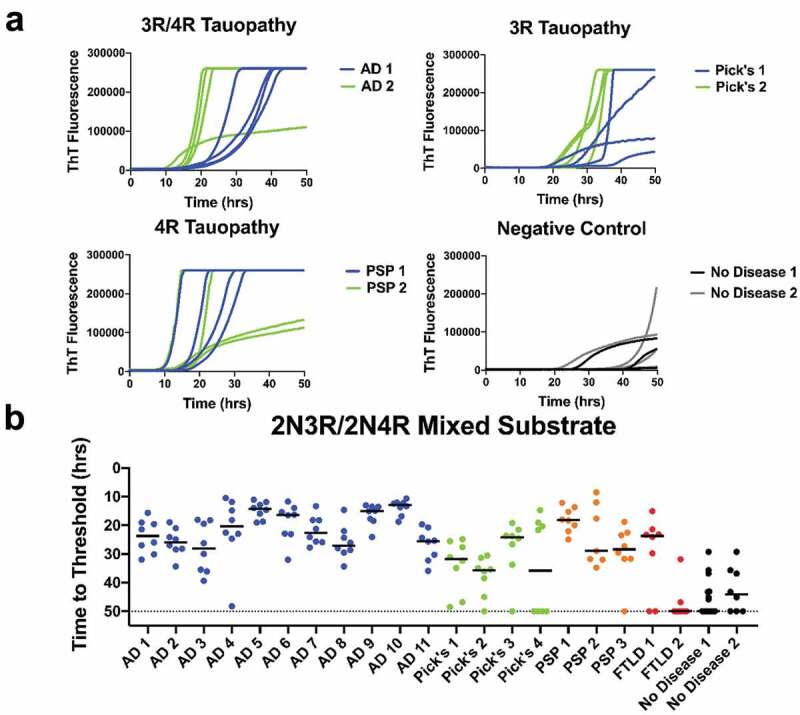
Tauopathy detection with 2N3R/2N4R substrate mixture. (a) ThT fluorescence curves for AD, Pick’s, PSP and no-disease brain controls using a 50:50 molar mix of 2N3R/2N4R substrate. (b) Time to threshold for 11 AD, 4 Pick’s, 3 PSP, and 2 FTLD brains and 2 no-disease controls in 2N3R/2N4R substrate, 1:100 brain dilution. Eight replicates for each sample are shown. Combined 3 R-4 R r-tau substrate produced the best compromise of sensitivity and selectivity vs. age-appropriate no-disease control brains

In an effort to improve sensitivity and specificity of the seeding assay, we examined a 50:50 molar ratio mixture of 2N3R and 2N4R tau substrates. The 2N3R/4 R substrate yielded threshold crossing times intermediate between the 2N3R and 2N4R substrates (Figures 2(b), 3(b), and 4(b)), produced ThT emission curves with smaller variance among replicates (Figure 4(a)), and reduced the spontaneous conversion background associated with the 2N3R only substrate, while not compromising selectivity (Figure 4(b)). These effects resulted in more selective detection of tauopathies compared with non-diseased samples (except for Pick’s 4 and FTLD2 samples) (Figure 4(b)). Overall, the mean rate of amyloid formation in AD and PSP samples was significantly different from that of negative control brains at *p* < 0.0001 and *p* < 0.001, respectively (Figure 4(b)).

### Detection of tau seeding in tauopathy mouse brains

**Figure 5.**
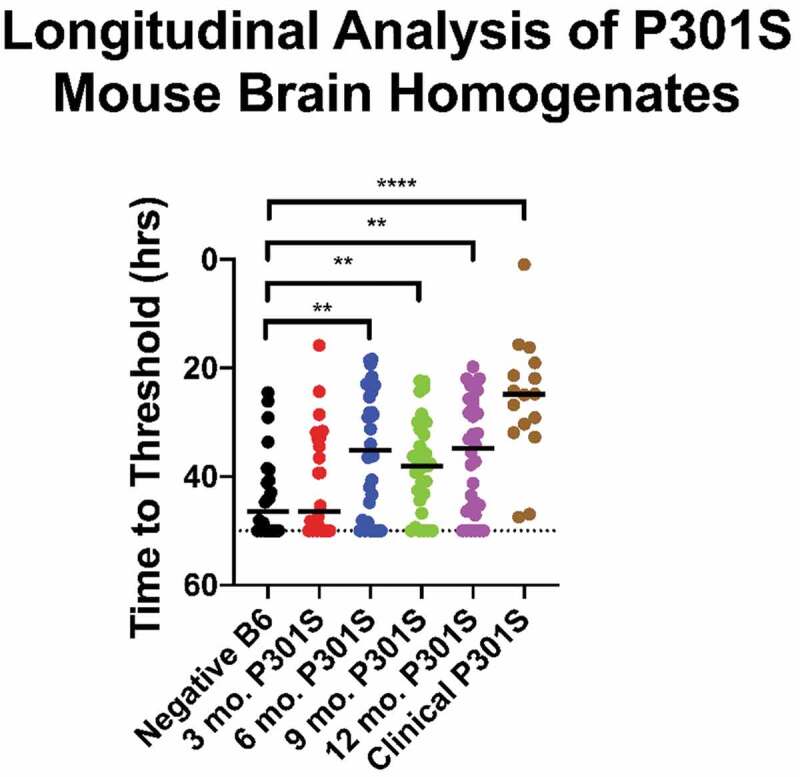
Preclinical detection of tauopathy in mice. Tau seeding activity was detected in brains of P301S mice collected at 3, 6, 9, and 12-months of age using the equimolar 2N4R/2N3R r-tau substrate. Data shown are based on 3–4 mouse pooled mouse brains at each timepoint. Y axis = time to threshold crossing

We assessed the tau 2N4R/2N3R tau substrate in brains of P301S B6 tauopathy pre-disposed mice expressing the 1N4R human tau gene with the P301S point mutation [24]. Cohorts of mice were euthanized according to pre-determined schedule of 3, 6, 9, or 12 months of age. Most of the mice analysed were in stage 1 disease, characterized by hind-limb grasping without weight loss or limb weakness [24]. Some mice euthanized at 12 months had progresses to stage 4 disease (clinical signs included weight loss, hunched back, and hind leg paralysis). Brain samples were prepared and tested using the 2N4R/2N3R tau substrate and the same preparation and RT-QuIC reaction conditions used for the above human samples. Three to four mouse brains were tested from each age cohort along with two clinical mouse brains as positive controls and B6 non-transgenic mice as negative controls. These assays demonstrated statistically significant differences in tau seeding in the brains of P301S mice euthanized at 6, 9, or 12 months compared with the B6 control mouse cohort (Figure 5). Brains from clinically affected mice produced shorter times to threshold compared with brains from asymptomatic or mildly symptomatic mice (Figure 5).

## Discussion

In the interest of contributing to the search for sensitive indicators to detect neurodegenerative diseases, we explored the use of the RT-QuIC seeding assays using full length recombinant tau substrates [25]. We observed seeding activity in AD and other tauopathy brain samples vs. a limited number of non-disease, age-appropriate human brain samples using 2N3R, 2N4R, or 2N3R/2N4R r-tau substrate mixture (Figures 2(b), 3(b), and 4(b)). We found the equi-molar 2N3R/2N4R r-tau mixture to produce the best combination of sensitive and specific detection with both 2N3R and 2N3R/2N4R mixed tau substrates.

It has been previously shown that a limitation in tauopathy detection is a strain-like variance imparted by the misfolded isoform(s) involved [19,20,22,23]. While we did not see seed-specific detection of tauopathy, as reported by Metrick and colleagues [22,23], using full length substrates we were able to detect seeding activity in 3 R, 4 R, or a 3 R/4 R tauopathy brains diluted up to 1000 fold (Figure 1(b)). This later finding supports those previously reported by of Weismiller et al. [26] who demonstrated that the 3 R/4 R tau cross-seeding barrier was reduced when full-length tau (htau40) was used [26]. However, we also found that detection was more selective when substrate and disease isoform were matched, or alternatively, a 3 R/4 R mixture was used (Figures 2(b), 3(b) and 4(b)). We do acknowledge that a significant limitation in conclusions possible in these studies is imposed by the small cohort of non-disease brains available to us.

We detected tau seeding activity prior to the onset of clinical symptoms in longitudinally sacrificed P301S mice. This would appear not to precede the detection of phosphorylated insoluble tau or hippocampal synapse loss in P301S mice as described by Lee and other investigators [27–31] (Figure 5). However, both of the above findings reinforce the basis for use of tau as a preclinical marker of neurodegenerative disorders. Here we demonstrate that the use of full-length tau substrates or mixture thereof can detect multiple tauopathies in hope that these findings will contribute to the further development of seeded amplification approaches for early detection of AD and other tauopathies.

## Materials and methods

### Tau preparation and purification

Full length 2N4R and 2N3R tau constructs were synthesized (Invitrogen) with point mutations changing cysteine to serine at residues 291 and 322 for 2N4R tau and 322 for 2N3R tau. Tau constructs were then placed in the pET 100 plasmid (Invitrogen) with a histidine tag. Recombinant 2N4R and 2N3R Tau was expressed in BL21 *Escherichia coli* (Novagen). Bacteria were cultured until the optical density at 600 nm (OD_600_) was 0.5–0.7 and expression was induced with 100 mM IPTG (sigma) for 3 hours. Cells were harvested by centrifugation and resuspended in 50 mM Na_2_HPO_4_, pH 6.8, 1 mM PMSF, HALT protease inhibitors (Pierce) and frozen overnight. The cell suspension was thawed, and cells were lysed by 3 rounds of sonication for 1 minute followed by a 5-minute rest on ice. The lysed cells were then boiled for 15 minutes and centrifuged at 12,000 rpm for 20 minutes. The supernatant was filtered through a 0.45 µM filter and applied to a His Trap HP (GE) column, equilibrated in 50 mM imidazole and 50 mM Na_2_HPO_4,_ pH 6.8, and eluted with an increasing gradient of imidazole to 0.5 M. Fractions with protein were collected and run through a HiPrep (GE) desalting column in buffer A (50 mM NaCl and 50 mM Na_2_HPO_4,_ pH 6.8) and were pooled. The pooled protein was finally loaded onto a HiTrap SP (GE) in the presence of buffer A and tau was eluted off of the column with a linear gradient of NaCl. Fractions containing the protein were collected, filtered with a 0.22 µm filter and a BCA assay (Pierce) was used to determine protein concentration. Protein was diluted to 30 µM concentration and stored at 4℃ until use.

### Sample preparation

Diseased and healthy human brain samples (Banner Sun Health Research Institute Brain and Body Donation Program, Sun City, AZ and New York University Alzheimer’s Disease Centre) were homogenized in phosphate buffer solution (150 mM NaCl, 20 mM Na_2_HPO_4_, pH 7.4) at a 10% w/v with protease inhibitors (sigma). Samples consisted of parts of occipital lobe or frontal cortex in brains with tauopathy categorized as Braak stage V–VI [32]. There was no detectable difference in seeding activity between the different brain regions. Fifty millilitres of the brain homogenates were spun for 2 minutes at 5000 rpm, the supernatant was discarded, and the pellet subjected to 2 rounds of ethanol precipitation treatment. For ethanol precipitation, pellets were resuspended in 100 µl 100% ethanol for 5 minutes and spun at 14,000 rpm for 5 minutes, and the supernatant was discarded. After 2 rounds of ethanol treatment, the pellets were washed in 100 µl PBS and spun at 14,000 rpm for 5 minutes. The resultant pellet was resuspended in N2 in PBS to the appropriate dilution.

All animal studies were approved by Colorado State University Institutional Animal Care and Use Committee (IACUC) and animals were handled in accordance with the protocols. Control B6 and B6; C3-Tg(Prnp-MAPT*P301S)PS19Vle/J hemizygous mice were purchased (Jackson laboratories) and mated. P301S hemizygous mice were genotyped and euthanized at 3, 6, 9, and 12 months of age to assess disease progression. Mouse brains were collected and frozen at −80℃ until use. P301S mice that progressed to stage 4 clinical status, as determined by two individuals, before 12 months of age were euthanized and their brains collected and frozen. Mouse clinical stages were reported from 0 to 4 with Stage 0 showing no signs of abnormal behaviour. Stage 1 disease was characterized as hind limb grasping when lifted by the tail. Most mice exhibited this trait as early as 3 months of age. Stages 2–3 included an increase in motor deficit defined by hind limb weakness and paresis. Stage 3 mice also exhibited weight loss. Stage 4 disease consisted of hind-limb paralysis, increased weight-loss and a hunched posture. Only clinical mice reached Stage 4 disease and all other time-point collected mice were scored as Stage 1. Mice seemed to progress from Stage 2 to 4 within weeks, so euthanasia and tissue collection occurred at clinical stage (Stage 4) for all mice exhibiting Stage 2 or above symptoms. For testing, a sagittal section of mouse brain was homogenized in PBS at a 10% w/v with protease inhibitors. Mouse brains were treated the same as human brains includ-ing an initial 2-minute spin to separate the pellet and supernatant followed by ethanol precipitation and resuspension in N2 in PBS.

### RT-QuIC tau assay

RT-QuIC experiments were carried out in black, optical-bottom 96-well (Nunc) plates with each well containing 20 µM recombinant Tau substrate, 15 mM Tris 200 67 mM NaCl, 0.83 mM KCl, 10 µM Heparin (sigma) and 1 mM Thioflavin T. Two microlitres of the sample mixture were added to each well of the plate. RT-QuIC experiments were carried out in a BMG Labtech Polarstar fluorometer/plate reader, with each cycle consisting of 1 minute of shaking at 700 rpm using a double-orbital setting followed by one minute of rest for 15 minutes. Fluorescence readings with an excitation of 450 nm and emission of 480 nm were conducted at the end of each 15-minute rest/shake cycle. The plate was read with 20 flashes per well with an orbital average of 4. Experiments consisted of 200 cycles (2N3R substrate and 2N3R/2N4R substrate mix) or 400 cycles (2N4R substrate) of fluorescent reading and an experimental well was deemed positive if the fluorescence in the well surpassed 20 standard deviations of the mean baseline of the wells. The time it took a sample to become positive was determined by the time-to-threshold calculator in the BMG Mars software. A Mann-Whitney T-Test was used to determine if samples were positive compared to the negative brain controls.

## References

[1] Consensus recommendations for the postmortem diagnosis of Alzheimer’s disease. The National Institute on Aging, and Reagan Institute Working Group on diagnostic criteria for the neuropathological assessment of Alzheimer’s disease. Neurobiol Aging. 1997;18(4 Suppl):S1–2. Epub 1997/07/01. PubMed PMID: 9330978. 9330978

[2] Blennow K, Vanmechelen E, Hampel H. CSF total tau, Aβ42 and phosphorylated tau protein as biomarkers for Alzheimer’s disease. Mol Neurobiol. 2001;24:87–97. 11831556 10.1385/MN:24:1-3:087

[3] Goedert M, Klug A, Crowther RA. Tau protein, the paired helical filament and Alzheimer's disease. Journal of Alzheimer's Disease. 2006 Jan 1;9(s3):195–207 10.3233/jad-2006-9s323 16914859

[4] Jakes R, Novak M, Davison M, et al. Identification of 3- and 4-repeat tau isoforms within the PHF in Alzheimer’s disease. Embo J. 1991;10(10):2725–2729. 1915258 10.1002/j.1460-2075.1991.tb07820.x PMC452980

[5] Mandelkow E-M, Mandelkow E. Biochemistry and cell biology of tau protein in neurofibrillary degeneration. Cold Spring Harb Perspect Med. 2012;2(7):a006247–a. 22762014 10.1101/cshperspect.a006247 PMC3385935

[6] Goedert M, Spillantini MC, Rutherford D, et al. Multiple lsoforms of human microtubule-associated protein tau: sequences and localization in neurofibrillah tangles of Alzheimer’s disease is found. Neuron. 1989;3(Table 1):519–526. 2484340 10.1016/0896-6273(89)90210-9

[7] Horie K, Barthélemy NR, Mallipeddi N, et al. Regional correlation of biochemical measures of amyloid and tau phosphorylation in the brain. Acta Neuropathol Commun. 2020;8(1):149. PubMed PMID: 32854776 32854776 10.1186/s40478-020-01019-z PMC7450927

[8] Fitzpatrick AW, Falcon B, He S, et al. Cryo-EM structures of tau filaments from Alzheimer’s disease. Nature. 2017;547(7662):185–190. . 28678775 10.1038/nature23002 PMC5552202

[9] Goedert M, Eisenberg DS, Crowther RA. Propagation of tau aggregates and neurodegeneration. Annu Rev Neurosci. 2017;40(1):189–210. PubMed PMID: 28772101 28772101 10.1146/annurev-neuro-072116-031153

[10] Atarashi R, Satoh K, Sano K, et al. Ultrasensitive human prion detection in cerebrospinal fluid by real-time quaking-induced conversion. Nat Med. 2011;17(2):175–178. .PubMed PMID: 21278748 21278748 10.1038/nm.2294

[11] Mays CE, Titlow W, Seward T, et al. Enhancement of protein misfolding cyclic amplification by using concentrated cellular prion protein source. Biochem Biophys Res Commun. 2009;388(2):306–310. PubMed PMID: 19664595; PubMed Central PMCID: PMC2756978 19664595 10.1016/j.bbrc.2009.07.163 PMC2756978

[12] Orru CD, Groveman BR, Hughson AG, et al. Rapid and sensitive RT-QuIC detection of human Creutzfeldt-Jakob disease using cerebrospinal fluid. mBio. 2015;6(1). DOI:10.1128/mBio.02451-14. PubMed PMID: 25604790; PubMed Central PMCID: PMC4313917 PMC4313917 25604790

[13] Sano K, Satoh K, Atarashi R, et al. Early detection of abnormal prion protein in genetic human prion diseases now possible using real-time QUIC assay. PLoS One. 2013;8(1):e54915. PubMed PMID: 23372790; PubMed Central PMCID: PMC3556051 23372790 10.1371/journal.pone.0054915 PMC3556051

[14] Zanusso G, Monaco S, Pocchiari M, et al. Advanced tests for early and accurate diagnosis of Creutzfeldt-Jakob disease. Nat Rev Neurol. 2016;12(6):325–333. .PubMed PMID: 27174240 27174240 10.1038/nrneurol.2016.65

[15] Bongianni M, Ladogana A, Capaldi S, et al. α-Synuclein RT-QuIC assay in cerebrospinal fluid of patients with dementia with Lewy bodies. Ann Clin Transl Neurol. 2019;6(10):2120–2126. 31599499 10.1002/acn3.50897 PMC6801172

[16] Candelise N, Schmitz M, Llorens F, et al. Seeding variability of different alpha synuclein strains in synucleinopathies. Ann Neurol. 2019;85(5):691–703. 30805957 10.1002/ana.25446

[17] Fairfoul G, McGuire LI, Pal S, et al. Alpha-synuclein RT-QuIC in the CSF of patients with alpha-synucleinopathies. Ann Clin Transl Neurol. 2016;3(10):812–818. 27752516 10.1002/acn3.338 PMC5048391

[18] Groveman BR, Orrù CD, Hughson AG, et al. Rapid and ultra-sensitive quantitation of disease-associated α-synuclein seeds in brain and cerebrospinal fluid by αSyn RT-QuIC. Acta Neuropathol Commun. 2018;6(1):7. 29422107 10.1186/s40478-018-0508-2 PMC5806364

[19] Kraus A, Saijo E, Metrick MA 2nd, et al. Seeding selectivity and ultrasensitive detection of tau aggregate conformers of Alzheimer disease. Acta Neuropathol. 2019;137(4):585–598. Epub 2018/12/21. PubMed PMID: 30570675; PubMed Central PMCID: PMCPMC6426988. 30570675 10.1007/s00401-018-1947-3 PMC6426988

[20] Metrick MA, Do Carmo Ferreira N, Saijo E, et al. Million-fold sensitivity enhancement in proteopathic seed amplification assays for biospecimens by Hofmeister ion comparisons. Proc Nat Acad Sci. 2019;116(46):23029–23039. 31641070 10.1073/pnas.1909322116 PMC6859373

[21] Metrick MA, Ferreira N, Saijo E, et al. A single ultrasensitive assay for detection and discrimination of tau aggregates of Alzheimer and Pick diseases. Acta Neuropathol Commun. 2020;8(1):22. 32087764 10.1186/s40478-020-0887-z PMC7036215

[22] Saijo E, Metrick MA, Koga S, et al. 4-Repeat tau seeds and templating subtypes as brain and CSF biomarkers of frontotemporal lobar degeneration. Acta Neuropathol. 2020;139(1):63–77. 31616982 10.1007/s00401-019-02080-2 PMC7192393

[23] Saijo E, Ghetti B, Zanusso G, et al. Ultrasensitive and selective detection of 3-repeat tau seeding activity in Pick disease brain and cerebrospinal fluid. Acta Neuropathol. 2017;133(5):751–765. Epub 2017/03/16. PubMed PMID: 28293793. 28293793 10.1007/s00401-017-1692-z

[24] Takeuchi H, Iba M, Inoue H, et al. P301S mutant human tau transgenic mice manifest early symptoms of human tauopathies with dementia and altered sensorimotor gating. PLoS One. 2011;6(6):e21050. Epub 2011/06/24. PubMed PMID: 21698260; PubMed Central PMCID: PMCPMC3115982. 21698260 10.1371/journal.pone.0021050 PMC3115982

[25] Jack CR Jr., Bennett DA, Blennow K, et al. NIA-AA research framework: toward a biological definition of Alzheimer’s disease. Alzheimers Dement. 2018;14(4):535–562. Epub 2018/04/15. PubMed PMID: 29653606; PubMed Central PMCID: PMCPMC5958625. 29653606 10.1016/j.jalz.2018.02.018 PMC5958625

[26] Weismiller HA, Murphy R, Wei G, et al. Structural disorder in four-repeat tau fibrils reveals a new mechanism for barriers to cross-seeding of tau isoforms. J Biol Chem. 2018;293(45):17336–17348. 30242125 10.1074/jbc.RA118.005316 PMC6231118

[27] Yoshiyama Y, Higuchi M, Zhang B, et al. Synapse loss and microglial activation precede tangles in a P301S tauopathy mouse model. Neuron. 2007;53(3):337–351. 17270732 10.1016/j.neuron.2007.01.010

[28] Guo JL, Lee VM. Seeding of normal tau by pathological tau conformers drives pathogenesis of Alzheimer-like tangles. J Biol Chem. 2011;286(17):15317–15331. Epub 2011/03/05. PubMed PMID: 21372138; PubMed Central PMCID: PMCPMC3083182. 21372138 10.1074/jbc.M110.209296 PMC3083182

[29] Falcon B, Cavallini A, Angers R, et al. Conformation determines the seeding potencies of native and recombinant Tau aggregates. J Biol Chem. 2015;290(2):1049–1065. Epub 2014/11/20. PubMed PMID: 25406315; PubMed Central PMCID: PMCPMC4294473. 25406315 10.1074/jbc.M114.589309 PMC4294473

[30] Holmes BB, Furman JL, Mahan TE, et al. Proteopathic tau seeding predicts tauopathy in vivo. Proce Nat Acad Sci. 2014;111(41):E4376–E85. 10.1073/pnas.1411649111 PMC4205609 25261551

[31] Jackson SJ, Kerridge C, Cooper J, et al. Short fibrils constitute the major species of seed-competent tau in the brains of mice transgenic for human P301S tau. J Neurosci. 2016;36(3):762–772. Epub 2016/01/23. PubMed PMID: 26791207; PubMed Central PMCID: PMCPMC4719013. 26791207 10.1523/JNEUROSCI.3542-15.2016 PMC4719013

[32] Braak H, Braak E. Staging of alzheimer’s disease-related neurofibrillary changes. Neurobiol Aging. 1995;16(3):271–278. 7566337 10.1016/0197-4580(95)00021-6

